# A New Sensitive Method for the Detection of Mycoplasmas Using Fluorescence Microscopy

**DOI:** 10.3390/cells8121510

**Published:** 2019-11-25

**Authors:** Anna Ligasová, Markéta Vydržalová, Renata Buriánová, Lenka Brůčková, Renata Večeřová, Anna Janošťáková, Karel Koberna

**Affiliations:** 1 Institute of Molecular and Translational Medicine, Faculty of Medicine and Dentistry, Palacký University Olomouc, Hněvotínská 5, 779 00 Olomouc, Czech Republic; renata.burianova@upol.cz (R.B.); Anna.Janostakova@fnol.cz (A.J.); 2 Faculty of Chemical Technology, University of Pardubice, Studentská 573, 532 10 Pardubice, Czech Republic; marketa.vydrzalova@upce.cz (M.V.); lenka.bruckova@upce.cz (L.B.); 3 Department of Microbiology, Faculty of Medicine and Dentistry, Palacký University Olomouc, Hněvotínská 3, 779 00 Olomouc, Czech Republic; Renata.Vecerova@fnol.cz

**Keywords:** cell cultures, mycoplasma infection, immunofluorescence detection

## Abstract

Contamination of cell cultures by mycoplasmas is a very common phenomenon. As they can substantially alter cell metabolism and potentially spread to all cell cultures in laboratory, their early detection is necessary. One of the fastest and cheapest methods of mycoplasma detection relies on the direct staining of mycoplasmas’ DNA by DAPI or Hoechst dyes. Although this method is easy and fast to perform, it suffers from the low signal provided by these dyes compared to the nuclear DNA. Therefore, the reporter cell lines are used for cultivation of mycoplasmas before DAPI or the Hoechst staining step. In the study presented, we have developed and tested a new immunofluorescence assay for the detection of mycoplasmas. The method is based on the enzymatic labeling using DNA polymerase I and modified nucleotides utilizing nicks in the mycoplasmas’ DNA. Modified nucleotides are incorporated into mycoplasmas’ DNA and subsequently visualized by immunofluorescence microscopy. The developed approach is independent of the mycoplasma strain, does not intensely stain nuclear DNA, does not stain other bacteria, and provides higher sensitivity than the approach based on the direct labeling using DAPI or Hoechst dyes.

## 1. Introduction

Mycoplasmas are wall-less bacteria [1]. It is presumed that the lack of the cell wall and also of some enzymatic activities and metabolic pathways is the result of degenerative evolution. Consequently, mycoplasmas are dependent on the nutrition substances contained in the surrounding environment [2]. Mycoplasmas are the smallest independently living organisms that are able to replicate on their own. Their genome’s size is 580–1358 kb [3]. The diameter of the cells is approximately 300–800 nm. The average generation time is around 1–3 h [4,5]. Presently, it is believed that more than 100 mycoplasma species exist [6]. Some species live as commensals on human oral and genital mucosa. Mycoplasmas can also cause several diseases such as pneumonia (*Mycoplasma pneumoniae*), inflammation of the urinary tract, or inflammation in the genital area (*Ureaplasma species*, *Mycoplasma genitalium* or *Mycoplasma hominis*) [7]. Although mycoplasmas were first isolated from humans in 1937, they were not cultivated on the selective medium until 1951 [8].

Mycoplasmas are among the microorganisms that frequently contaminate eukaryotic cell cultures as well. Cell cultures can be contaminated in several ways. The most frequent ways are the use of contaminated cultivation solutions, the transfer of contamination from another contaminated cell line, or contamination from the laboratory personnel [4,8,9]. In the case of primary cell cultures, the source of contamination can be the original tissue as well [10]. As mycoplasmas are small organisms with relatively flexible cell membrane, they can pass through the commonly used 0.45 µm microbiological filters [4]. The contamination of cell lines is mainly caused by the following species: *Mycoplasma arginini*, *Mycoplasma fermentas*, *Mycoplasma hyorhinis*, *Mycoplasma orale*, *Mycoplasma hominis,* and *Acholeplasma laidlawii* [4,8,10]. The cell line contamination is often hidden even at high mycoplasma densities as the turbidity of the culture medium or the change of the pH of the medium is not usually visible [5,9]. Furthermore, mycoplasmas are resistant to most of the commonly used antibiotics [9].

Mycoplasmas exhibit various effects on cell lines. The majority of the changes are caused by the competition of cells and mycoplasmas for nutritional substances and by the products of mycoplasma metabolism [4,5,11]. In this respect, mycoplasmas gain energy from the fermentative metabolism of various nutrients leading to the metabolites toxic for eukaryotic cells [11]. Mycoplasma contamination has an impact on cell growth, amino acids’ metabolism, the induction of chromosomal aberrations, induction or inhibition of lymphocyte activation, induction or suppression of cytokines, or even an impact on signal transduction [4,5,11]. Mycoplasmas are also dependent on the uptake of cholesterols, sterols, and lipids, leading to changes in the composition of cell membranes [11]. Moreover, the impact of mycoplasmas is not the same for different cell types [4].

Currently, several methods are available for the detection of mycoplasma presence in cell culture. Each of them has its advantages and disadvantages from the point of view of the speed, specificity, sensitivity, and overall costs. Basically, it is possible to divide them into several groups [4]:

- histological staining
- electron microscopy detection
- biochemical methods (e.g., assays based on enzyme activity measurement)
- immunological methods (e.g., ELISA or fluorescence detection in situ)
- staining by DNA dyes (DAPI, Hoechst 33258)
- microbiological approaches (agar/broth media assay)
- hybridization (e.g., filter or liquid hybridization)
- polymerase chain reaction (PCR) and real-time PCR

For the reliable revelation of mycoplasma presence, at least two different approaches are recommended to be simultaneously used for testing the samples. In the case of laboratories producing various commercial biological products, two main methods are presently accepted. They are described in the national and international regulatory documents. The first method is the agar/broth media assay, the second is the indicator cell culture assay (also called indirect DAPI staining) [2]. In the case of the agar/broth media assay, only cultivable mycoplasmas are detected (mycoplasmas able to grow on the used medium). Detection relies on the occurrence of mycoplasma colonies on the surface of the medium. The second approach detects also fastidious (or uncultivated) mycoplasmas. In this case, reporter mammalian cells are used (e.g., Vero cells or NIH 3T3 or 3T6 cells) which support the growth of mycoplasmas. Reporter cells are incubated for at least three days with the culture medium from the tested cells. Then, mycoplasmas’ DNA is stained using DAPI of Hoechst dyes [2,4,6]. The disadvantage of both approaches is the long testing time (circa 28 days in the case of the agar/broth media assay and circa 10 days in the case of the indicator cell culture assay). Moreover, the sensitivity of the agar/broth media assay can be influenced by the preparation of the medium and the quality of the particular components of the medium. The results of the tests may be also influenced by handling with the mycoplasma samples [2,6]. The reason for such a long cultivation of samples in the case of DAPI or Hoechst staining is the low signal provided by DNA stains and the subsequent problems with the interpretation of the results in the case of a low mycoplasma density. In the case of contamination of other bacteria, extranuclear fluorescent signals produced by these bacteria hinder the clear interpretation of the results [12]. Further, the signal from the nuclear area make the resolution of mycoplasmas more difficult [13]. The DAPI or Hoechst dyes are not as convenient dyes for the direct observation as e.g., fluorochromes with the excitation wavelengths in the green area of the light due to the low sensitivity of human eyes for DAPI or Hoechst (emission wavelength is ca 460 nm), as seen in reference [14]. Although it can be overcome by the use of sensitive cameras, the part of the signal is still masked by a nuclear signal. Moreover, the most common cameras available on the market have higher sensitivity outside of the DAPI/Hoechst region [15,16].

In the study presented, we have developed a new assay for the detection of mycoplasmas present in cell cultures. The approach is based on the enzymatic labeling of mycoplasmas’ DNA using modified nucleotides followed by their subsequent detection using specific antibodies. Contrary to the method based on DAPI or Hoechst staining, the developed method does not intensively label nuclear DNA and enables users to choose the fluorochrome to match their needs and consequently, it allows a much clearer interpretation of the obtained results, particularly in the case of the low density of mycoplasmas.

## 2. Materials and Methods

### 2.1. Cell Cultures

A549 (human lung carcinoma cells, purchased from ECACC, UK) and Lep cells (human diploid fibroblasts, purchased from Klinlab s.r.o.) were used during the development of the approach. A549 cells were grown in Minimum Essential Medium Eagle with *L*-glutamine and sodium bicarbonate in the presence of 10% fetal calf serum, 1 mM pyruvate, 10 mM HEPES, 50 µg/mL penicillin, and 50 µg/mL streptomycin (all supplements were from Life Technologies, USA). The Lep cells were cultivated in Dulbecco’s modified Eagle’s medium (DMEM, Gibco) supplemented with 20% fetal bovine serum (PAA Laboratories), 3.7 g/L of sodium bicarbonate (Sigma Aldrich), and 50 µg/mL of gentamicin (Lek Pharmaceuticals, Ljubljana, Slovenia).

The cells were grown on coverslips (12 mm in diameter) in Petri dishes at 37 °C in a humidified atmosphere containing 5% CO_2_.

### 2.2. Mycoplasma Species, Inoculation of Cell Line with the Mycoplasma Strains

We used the following mycoplasma strains: *Mycoplasma hominis* (ATCC 23114), *Mycoplasma fermentans* (ATCC 49891), and *Mycoplasma arginini*. Mycoplasmas were cultured in PPLO broth (BD Difco™ Mycoplasma Broth, 255420) supplemented with 20% horse serum (TCS Biosciences, Buckingham, United Kingdom), 10% yeast extract, 0.5% l-arginine (Merck, Darmstadt, Germany), and indicator phenol red for 24 h at 37 °C in an atmosphere containing 5% CO_2_.

A549 cells were inoculated with *M. hominis* with the following concentrations: 1 × 10^6^ CFU/mL, 4 × 10^5^ CFU/mL, 2 × 10^5^ CFU/mL, 1 × 10^5^ CFU/mL, 5 × 10^4^ CFU/mL, 2.5 × 10^4^ CFU/mL, and 1.25 × 10^4^ CFU/mL (CFU = colony forming units). In the case of *M. fermentans* and *M. arginini*, the cells were inoculated with the concentration of 1 × 10^6^ CFU/mL. The cells were grown for 24 h at 37 °C in an atmosphere containing 5% CO_2_.

Lep cells were accidentally infected with the unknown mycoplasma strain. The mycoplasma infection was confirmed by RT-PCR, the developed approach (Supplementary Figure S1a,b), and a MycoAlert^TM^ PLUS Mycoplasma Detection Kit (East Port Praha s r. o. (Lonza, LT07-703), Prague, Czech Republic).

### 2.3. Bacterial Strains

Representatives from the group of Gram-positive and Gram-negative bacteria were tested. We used American Type Culture Collection strain *Escherichia coli* (ATCC 25922, *E. coli*). Strains *Staphylococcus epidermidis* CCM 7221 (*S. epidermidis)* and *Streptococcus salivarius* (*S. salivarius*) were obtained from the culture collection of the Department of Microbiology, Faculty of Medicine and Dentistry, Palacký University Olomouc. Bacteria were cultivated in the Mueller Hinton broth (BIORAD, Hercules, CA, USA) for 16 h at 37 °C.

All bacterial strains were deposited on the glass coverslips by wet cytocentrifugation (100× *g*, 5 min) using the CytoTrap adaptor (https://www.imtm.cz/sites/default/files/imtm_technologies_2019_07_16.pdf). *E. coli* was also deposited on coverslips with Lep cells accidentally infected with mycoplasma. Samples were fixed with 2% formaldehyde in 1× PBS for 5 min at room temperature (RT), centrifuged (100× *g*, 5 min), washed with 1× PBS, centrifuged again (100× *g*, 5 min), and coverslips were removed from the CytoTrap apparatus. Next, cells were permeabilized with 0.2% Triton X-100 (10 min, RT).

### 2.4. Mycoplasma Detection by the Developed Approach

The samples were fixed by formaldehyde, ethanol, or acetic acid along with methanol. In the case of formaldehyde fixation, the samples were incubated with 2% formaldehyde in 1× PBS for 10 min, washed three times with 1× PBS, and permeabilized with 0.2% Triton X-100 (10 min, RT). If ethanol was used, the then samples were incubated in ice-cold 70% ethanol (1 h, −20 °C). In the third case, the samples were incubated with an ice-cold solution of acetic acid and methanol (1:3) for 10 min at −20 °C [6]. After the fixation step, the samples were washed with 1× PBS if not stated otherwise and incubated in the enzymatic mixture.

During protocol optimization, we tested the following marker nucleotides: 5-(*N*-[*N*-biotinyl-ε-aminocaproyl-γ-aminobutyryl]-3-aminoallyl)-2′-deoxyuridine 5′-triphosphate (biotin-dUTP, Sigma Aldrich (Roche, 11093070910), Prague, Czech republic), digoxigenin-3-*O*-methylcarbonyl-ε-aminocaproyl-[5-(3-aminoallyl)-2-deoxy-uridine-5′-triphosphate (digoxigenin-dUTP, Roche) and ChromaTide^®^ fluorescein-12-dUTP (fluorescein-dUTP, ThermoFisher Scientific, Waltham, MA, USA). The composition of the enzymatic mixture is shown in Table 1. This composition was used if not stated otherwise. During optimization, we incubated the samples in the enzymatic mixture for 10, 20, 30, or 60 min at RT. A 30-min incubation time was used in the subsequent experiments. After incubation in the enzymatic mixture, the samples were washed in 1× PBS and the incorporated biotin or digoxigenin was detected. If fluorescein-dUTP was used, the samples were washed with 25 mM Tris-HCl, pH 7.5 and 150 mM NaCl (Tris-NaCl buffer) after incubation in the enzymatic mixture and mounted in the mounting medium composed of the solution of 90% glycerol, 50 mM Tris-HCl, pH 8.0, and 2.5% 1,4-diazabicyclo [2.2.2]octane (DABCO, Sigma Aldrich, Prague, Czech Republic).

Biotin-dUTP and digoxigenin-dUTP were visualized by indirect immunofluorescence using antibodies. Biotin-dUTP was alternatively visualized by fluorescently labeled streptavidin as well. In the case of indirect immunofluorescence, the samples were incubated with the rabbit anti-biotin antibody (1:100, Abcam, Cambridge, United Kingdom) or mouse anti-digoxigenin antibody (1:100, Roche, primary antibodies) diluted in a Tris-NaCl buffer for 30 min, washed three times with the Tris-NaCl buffer, and then incubated with the anti-rabbit or anti-mouse antibody conjugated with the fluorochrome Alexa Fluor 488 diluted in the Tris-NaCl buffer (1:100, Jackson ImmunoResearch, secondary antibodies) for 30 min. If the detection of biotin-dUTP was performed by fluorescently labeled streptavidin, the samples were incubated with streptavidin from *Streptomyces avidinii* conjugated with FITC (1:100, Sigma Aldrich, Prague, Czech Republic) diluted in the Tris-NaCl buffer for 30 min. After washing with the Tris-NaCl buffer, the samples were mounted in the mounting medium.

In some experiments, the mitochondrial marker MTCO2 (1:100, Abcam, Cambridge, United Kingdom) was added to the mixture of the primary antibody. In this case, DyLight 649 anti-mouse antibody (1:100, Jackson ImmunoResearch, Ely, United Kingdom) was added to the mixture of the secondary antibody. If DAPI was used, it was added to the mixture of the secondary antibody (the final concentration was 0.5 µg/mL). In the case of fluorescently labeled streptavidin or fluorescein-dUTP, samples were incubated with DAPI (0.5 µg/mL in Tris-NaCl buffer) for 30 min.

### 2.5. Nuclease Treatment

In some experiments, samples were incubated in the solution of DNase I and Exonuclease III before the enzymatic labeling. In this case, the nuclease solution contained DNase I (1 U/µL, the working concentration was 100 U/mL; one unit of DNase I completely degrades 1 µg of plasmid DNA in 10 min at 37 °C, ThermoFisher Scientific, EN0525), Exonuclease III (200 U/µL, the working concentration was 4 U/µL; one unit of Exonuclease III catalyzes the release of 1 nmol of acid soluble reaction products from *E. coli* [^3^H]-DNA in 30 min at 37 °C, ThermoFisher Scientific, EN0191), 1× buffer for Exonuclease III (ThermoFisher Scientific) and 0.1 mM CaCl_2_. The samples were incubated in the solution for 2 h at 37 °C. The control samples were left in 1× PBS.

### 2.6. Alternative Approaches of Mycoplasma Detection

#### 2.6.1. DAPI and Hoechst Staining

For the direct DAPI and Hoechst staining, the modified protocol of Young et al. was used [6]. Briefly, the samples were fixed using either formaldehyde or an acetic acid: methanol solution (see above). After washing in the Tris-NaCl buffer (DAPI) or distilled water (Hoechst 33258), the cellular and mycoplasmas’ DNA was stained for 10 min in the solution of 0.05 µg/mL DAPI in the Tris-NaCl buffer or in the solution of 0.05 µg/mL Hoechst 33,258 in distilled water. After washing the samples in the Tris-NaCl buffer (DAPI) or distilled water (Hoechst 33258), the samples were mounted in the mounting medium.

#### 2.6.2. PCR Detection

Cell lysates were prepared by the incubation of the cells in the lysis buffer (10 mM Tris-HCl pH 8.3, 50 mM KCl, 1% Triton X-100 and 100 µg/mL proteinase K; 2.5 × 10^5^–5 × 10^5^ cells were used per sample) at 37 °C overnight. Prior to the PCR reaction, proteinase K was inactivated by a 10-min incubation of the samples at 95 °C. 16S rRNA gene sequence specific primers and the specific Myco P probe (forward primer Myco 1, reverse primer Myco 2, fluorescent probe Myco P, all custom-made, Generi Biotech, for details see Table 2) were used. The used probe detects the following mycoplasma strains: *M. bovigenitalium*, *M. bovirhinis*, *M. bovis*, *M. californicum*, *M. citelli*, *M. columbianum*, *M. fermentans*, *M. hominis*, *M. hyorhinis*, *M. iners*, *M. lipophilum*, *M. meleagridis*, and *M. synoviae*.

The master reaction mix contained 1× Taq reaction buffer, 2 mM MgCl_2_, 0.8 µM Myco 1 Forward Primer, 0.8 µM Myco 2 Reverse Primer, 0.2 µM Myco P probe, 0.2 mM of the mixture of dNTPs (Promega), and 0.06 U/µL Taq DNA Polymerase (ThermoFisher Scientific, AB-0301B). Subsequently, 24 µL of the prepared master reaction mix was added per well of the 96-well plate. Then, one microliter of the tested sample per well was added to the master reaction mix. Besides the tested samples, positive and negative controls were also included. As the positive control, 1 µL of mycoplasma DNA isolated from the mycoplasma-positive cell line was used. As the negative control, 1 µL of the distilled water was used. The real-time PCR was performed using the parameters described in Table 3 using the LightCycler^®^ 480 (Roche).

The fluorescence signal was measured using a FAM channel (465 nm excitation and 510 nm emission). The results were automatically analyzed by the LightCycler^®^ 480 software (Roche Diagnostics, Prague, Czech Republic). Every sample with a fluorescence signal higher than the noise curve was considered as a mycoplasma positive sample.

#### 2.6.3. MycoAlert^TM^ PLUS Mycoplasma Detection Kit

The MycoAlert kit was used according to the manufacturer’s instructions (Lonza, LT07-710). Briefly, 2 mL of culture media from cells before trypsinization was centrifuged at 200× *g* for 5 min. 100 µL of the supernatant was transferred to the well of the white 96-well plate and 100 µL of MycoAlert™ PLUS Reagent was added. The samples were incubated for 5 min at RT and the luminescence was read (Reading A, 1 s, integrated reading, Tecan Plate reader). Then, 100 µL of MycoAlert™ PLUS Substrate was added and the samples were incubated for 10 min at RT. The luminescence was read (Reading B, 1 s, integrated reading, Tecan Plate reader). The ratio of the measured values was calculated according to the formula: Reading B/Reading A. The values under 0.9 were interpreted as mycoplasma negative, values above 1.2 as mycoplasma positive. Values between 0.9 and 1.2 are a borderline. One positive control (MycoAlert^TM^ Assay Control) and two negative controls (distilled water and culture medium) were used during testing.

### 2.7. Fluorescence Microscopy

The images were obtained using an Olympus IX83 microscope (UPLSAPO O objective 100×, NA 1.4) equipped with a Zyla camera (Andor) with a resolution of 2048 × 2048 pixels using acquisition software (CellSense Dimension, Olympus). If not stated otherwise, the images were acquired in the *Z* stack mode and the final images are presented as a projection of the maximal intensity of the *Z* stack.

### 2.8. Data Evaluation

The data were analyzed using CellProfiler image analysis software [17,18], Microsoft Excel and the final graph was made in GraphPad Prism 6 software. All the measurements were performed for three independent experiments. 100 images were analyzed per experiment. The data are presented as the mean values ± standard deviation (STD).

The standard curve of PCR results was done from the crossing points (also known as threshold cycles) [19] of particular mycoplasma concentrations using GraphPad Prism 6 software.

**Figure 1:**
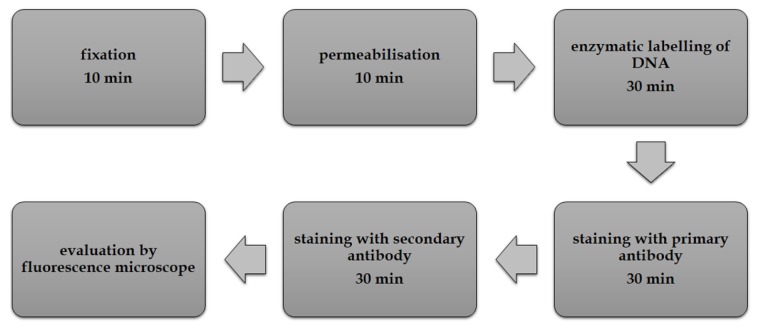
Scheme of the procedure. The scheme of the developed approach is shown. It does not include short washing steps between individual steps.

The scheme from Figure 1 was drawn using the online application (draw.io) and Adobe Photoshop CS4 software (Adobe, San Jose, CA, USA).

## 3. Results and Discussion

### 3.1. Method Description

The optimized approach consists of six steps performed at RT (Figure 1).

Brief washing steps using 1× PBS buffer or Tris-NaCl buffer are inserted among the particular steps. After fixation with 2% formaldehyde and permeabilization with 0.2% Triton X-100, the samples are incubated in a solution for enzymatic labeling containing biotin-dUTP. The solution contains also DNA polymerase I (Pol I) which can utilise a 3′-OH group in breaks in DNA and labels these sites by biotin-dUTP [20]. Surprisingly, in contrast to the nuclear or mitochondrial DNA, the mycoplasmas’ DNA visualization does not require the introduction of nicks or double stranded breaks in DNA [20,21]. If we induced the formation of breaks in DNA by monovalent copper ions [20], then we observed a high increase of the signal of nuclear and mitochondrial DNA, but not of mycoplasmas’ DNA. Instead, the signal decreased. Moreover, an increase of the signal in cell nuclei and mitochondria induced by copper ions obscured the mycoplasma-derived signal. In this respect, experiments done on the next day after cell fixation also showed that the signal in cell nuclei and mitochondria gradually increased. It indicates that the gradual degradation of DNA occurs during the sample storage in 1× PBS buffer. It was evident that the freshly fixed samples provide a higher ratio between the signal from mycoplasmas’ DNA and the signal derived from nuclei and mitochondria. After the incubation of the cells in the solution for enzymatic labeling containing biotin-dUTP, the incorporated modified nucleotides are visualized using indirect immunofluorescence. Anti-biotin antibody and fluorochrome-labeled antibody raised against the anti-biotin antibody is used during biotin visualization. Alternatively, digoxigenin-dUTP, anti-digoxigenin antibody and fluorochrome-labeled antibody raised against the anti-digoxigenin antibody can be used. If the endogenous biotin is present, the digoxigenin-dUTP can provide a higher signal/background ratio. Finally, the samples are evaluated by fluorescence microscopy. If required, it is possible to add DAPI or Hoechst stain to the solution of the secondary antibody. In the case of a low accruement of mycoplasmas and the use of samples with the damaged DNA e.g., after long storage, it can be helpful to add a mitochondrial marker. It makes it possible to eliminate the possible signal from the mitochondria.

The experiments with DNA nucleases clearly showed that the presence of mycoplasmas’ DNA is essential for the signal production by DNA polymerase I. In these experiments, A549 cells inoculated with 1 × 10^6^ CFU/mL of *M. hominis* were fixed with formaldehyde, permeabilized with Triton X-100 and incubated in the solution of DNase I and Exonuclease III for 2 h at 37 °C before the enzymatic labeling. The activity of nucleases led to the complete loss of the signal from the mycoplasmas’ DNA (Supplementary Figure S2).

The reason of the relatively high signal provided by DNA polymerase reaction in the case of mycoplasmas’ DNA but not in the case of cell nuclei is not clear. Some data indicates that mycoplasmas can contain cell membrane associated magnesium and calcium dependent nucleases and that their activity can be stimulated by the detergent addition [22]. Since at least part of these nucleases can be released from the membrane during the fixation/permeabilization step, it could result in the nick formation in mycoplasmas’ DNA. However, our experiments with the bivalent ion chelator—ethylenediaminetetraacetic acid (EDTA), showed that the addition of EDTA to the fixation and/or permeabilization solutions did not result in the signal lowering. This indicates that the mycoplasmas’ DNA is not the target of the membrane associated nuclease attack during the fixation/permeabilization step. Instead, it seems that these nicks are formed before the fixation/permeabilization step or the nuclease activity is independent of bivalent ions. As mycoplasmas contain a very limited number of genes, their metabolism, DNA organization, and DNA repair is highly different from the metabolism, organization of DNA, and DNA repair of mammalian cells, and even of other bacteria [23]. We suppose that these differences result in a higher rate of nick formation and/or nick accessibility for DNA polymerase reaction.

### 3.2. Procedure Optimisation

**Figure 2:**
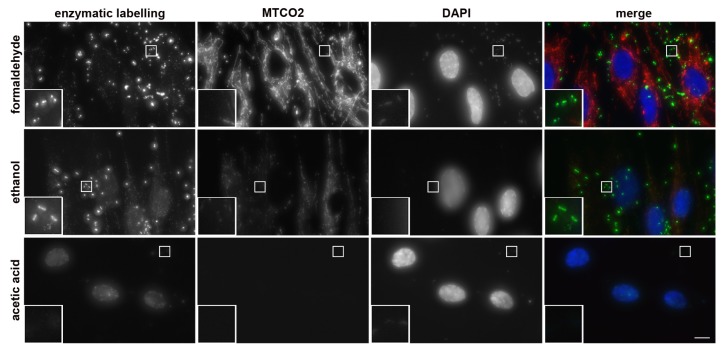
Effect of fixation protocol on the signal intensity. The results of the detection of mycoplasmas’ DNA in samples with Lep cells accidentally infected with mycoplasma (RT-PCR-positive, MycoAlert-positive) using the developed approach and three fixation protocols is shown. The samples were fixed either with formaldehyde, with 70% ethanol, or in a solution of acetic acid and methanol (1:3). The enzymatic mixture contained biotin-dUTP. Biotin-dUTP was detected by indirect immunofluorescence (green). The overall DNA was labeled by DAPI (blue), mitochondria were labeled with the mitochondrial marker MTCO2 (red). The images were acquired for 16.56 ms (biotin-derived signal); 2.636 ms (DAPI) and 7.916 ms (MTCO2-derived signal). Scale bar = 10 µm.

During the procedure’s development, we tested three fixation protocols. They were based on formaldehyde, ethanol, or a mixture of acetic acid and methanol. If ethanol fixation or acetic acid: methanol fixation was used, the permeabilization step was omitted. We found that the highest signal was provided by formaldehyde fixation (Figure 2). Ethanol fixation resulted in a decrease of the signal (Figure 2). The signal after acetic acid: methanol fixation was almost completely suppressed (Figure 2). No signal was observed if mycoplasma negative samples were used. It clearly indicated that the acetic acid: methanol solution is not an appropriate fixation protocol for mycoplasma detection using the developed approach.

More detailed analysis of the signal intensity in the samples fixed with ethanol or formaldehyde showed that the integral intensity of the mycoplasma-derived signal per image measured in ethanol-fixed cells was circa 69% of the signal measured in the formaldehyde-fixed cells (Supplementary Figure S3). Ethanol fixation also resulted in a substantial decrease of the signal of the mitochondrial marker (Figure 2). In this respect, the fixation protocol based on the acetic acid: methanol mixture resulted in the complete loss of the signal from the mitochondrial marker.

**Figure 3:**
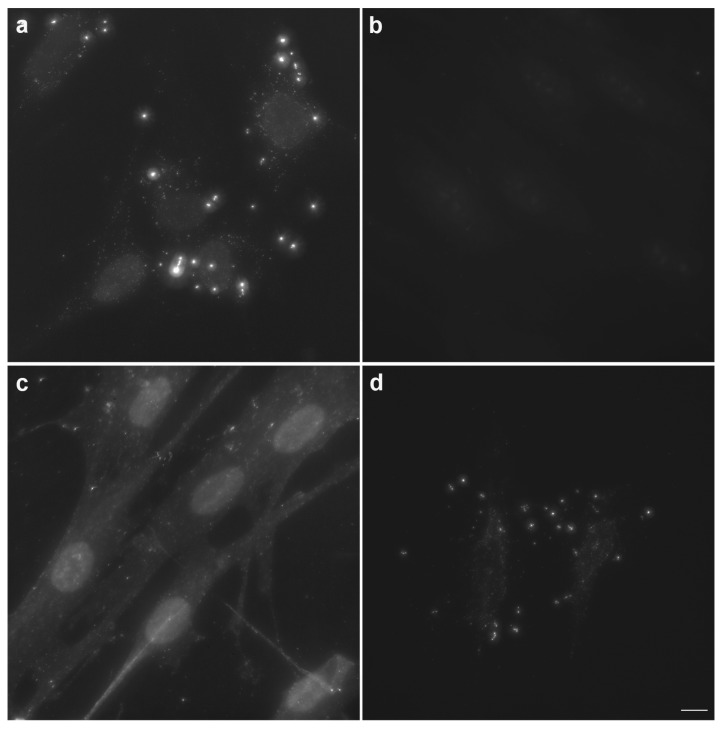
Effect of marker nucleotide, the detection approach and dTTP on the signal intensity. The samples containing Lep cells accidentally infected with mycoplasma were fixed by formaldehyde, permeabilized by Triton X-100 and mycoplasmas’ DNA was detected using enzymatic detection with Pol I. (**a**) The enzymatic mixture contained biotin-dUTP. Biotin-dUTP was visualized using a primary antibody (rabbit anti-biotin) followed by a secondary antibody (conjugated with Alexa Fluor 488). (**b**) The enzymatic mixture contained fluorescein-dUTP. (**c**) The enzymatic mixture contained biotin-dUTP. Biotin-dUTP was visualized by streptavidin conjugated with FITC. (**d**) The enzymatic mixture contained biotin-dUTP and an equimolar amount of dTTP. Biotin-dUTP was visualized using a primary antibody (rabbit anti-biotin) followed by a secondary antibody (conjugated with Alexa Fluor 488). The images were acquired for 11.44 ms. Scale bar = 10 µm.

The high signal provided by biotin-dUTP can be partially attributed to the high content of A:T pairs in the mycoplasmas’ DNA [2,10]. The average A:T content of mycoplasma genomes ranges from 60% to 79% [1]. The replacement of biotin-dUTP by digoxigenin-dUTP provided a similar signal to that in the case of biotin-dUTP. Surprisingly, the use of dUTP coupled with fluorescein instead of biotin-dUTP led to the nearly complete loss of the signal (compare Figure 3a,b and Supplementary Figure S4). The analysis of the signal in DAPI positive areas (outside the cell nucleus) containing a discernable fluorescein signal showed that the average signal/background ratio was circa 1.4 ± 0.2 in the case of fluorescein-dUTP. In the case of biotin- or digoxigenin-labeled dUTP, the ratio was ca 21.1 ± 3.3. In the case of DAPI, the ratio was circa 3.8 ± 0.7. The background was measured 1 µm apart from the border of the non-nuclear, signal positive, areas.

The substitution of the antibody-based detection system for biotin-dUTP visualization by the system based on FITC-labeled streptavidin resulted in the loss of the specific signal as well (Figure 3c). The addition of dTTP to the enzymatic mixture resulted in a decrease of the signal (Figure 3d).

**Figure 4:**
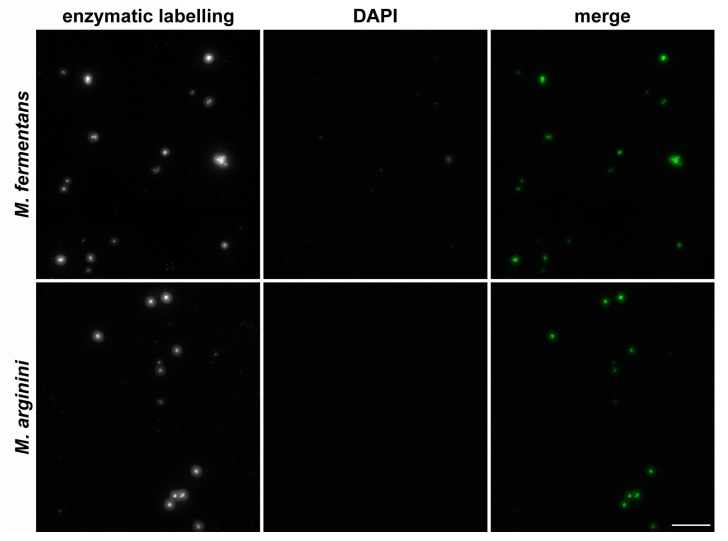
Detection of mycoplasmas by the developed approach and DAPI. Samples with A549 cells were inoculated with *M. arginini* (1 × 10^6^ CFU/mL) or *M. fermentans* (1 × 10^6^ CFU/mL). Mycoplasmas were detected using an enzymatic mixture containing biotin-dUTP followed by indirect immunofluorescence. Mycoplasmas’ DNA was simultaneously stained with DAPI. The images were acquired for 2.636 ms (*M. fermentans*) and for 3.788 ms (*M. arginini*). Scale bar = 10 µm.

The example of the detection of two mycoplasma strains (*M. arginini* and *M. fermentans*) by the developed approach and DAPI is shown in Figure 4.

In summary, the obtained results showed that the most suitable variant of the enzymatic labeling of mycoplasmas’ DNA is the protocol based on formaldehyde fixation followed by permeabilization with Triton X-100. Biotin -dUTP can be visualized by indirect immunofluorescence, but not by streptavidin or by digoxigenin-dUTP followed by indirect immunofluorescence.

### 3.3. Labeling of Bacterial Cells Using the Developed Approach

**Figure 5:**
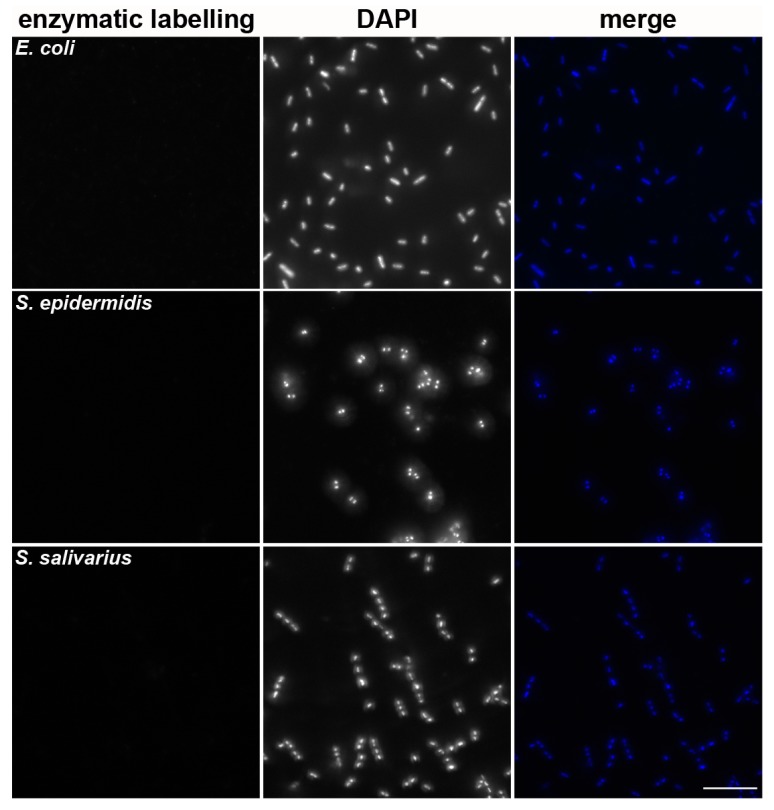
Testing of the developed approach for the detection of bacterial cells. *E. coli*, *S. epidermidis,* and *S. salivarius* deposited on the glass coverslips were fixed by formaldehyde, permeabilized by Triton X-100 and stained using the developed approach (biotin-dUTP) and DAPI. Scale bar = 10 µm.

We tested whether the developed approach also detects bacteria with the cell wall. We used the following bacterial strains: *E. coli*, *S. epidermidis*, and *S. salivarius*. Bacterial cells were deposited on coverslips using CytoTrap apparatus and wet cytocentrifugation to protect the cells from drying. In the control experiments, *E. coli* was deposited on the coverslips with Lep cells accidentally infected with mycoplasmas. Coverslips with the captured bacteria or mycoplasma-infected Lep cells with *E. coli* were processed according to the developed approach. All samples were stained with DAPI as well. It was obvious that the developed approach does not stain the bacterial cells (Figure 5 and Supplementary Figure S5).

### 3.4. Comparison of the Developed Approach with the Direct DAPI or Hoechst Staining

Although DAPI exhibits a preference for A:T rich sequences of DNA as well [24], only a very weak DAPI signal was observed in the non-nuclear areas of mycoplasma positive samples under the acquisition conditions that result in the saturation of the signal in less than 5% of the area occupied by cell nuclei in the formaldehyde-fixed cells compared to for the biotin-dUTP derived signal (Figure 2). The other two fixation protocols provided a lower signal under these acquisition conditions. As the low or missing signal could result from the fact that the samples were treated by the developed procedure, we performed DAPI staining of the enzymatically non-treated cells. However, we did not observe any impact of the omission of enzymatic step on the signal.

**Figure 6:**
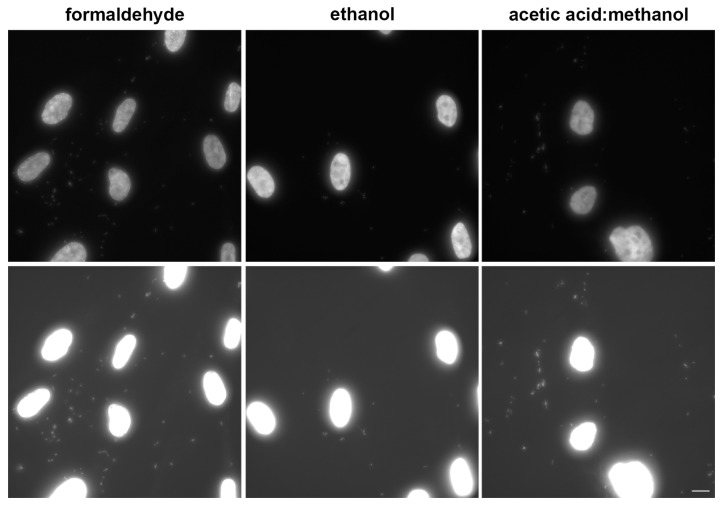
The effect of the fixation protocols on the DAPI staining of mycoplasmas in the samples containing Lep cells accidentally infected with mycoplasma is shown. The cells were fixed either with formaldehyde or with ethanol or in a solution of acetic acid and methanol. The fixed cells were stained with 0.05 µg/mL DAPI for 10 min. The upper images were acquired for 19.916 ms (formaldehyde- and ethanol-fixed cells) or 11.444 ms (acetic acid: methanol-fixed cells). The lower images were acquired for circa six-fold longer times. Scale bar = 10 µm.

We also tested the impact of the 10-fold lower concentration of DAPI as the higher content of A:T pairs in mycoplasmas’ DNA compared to the nuclear DNA can result in an increase of the ratio between the mycoplasmas’ DNA and the nuclear DNA signals. The concentration decrease, however, did not provide a higher ratio between mycoplasmas’ and nuclear DNA in the samples of Lep cells accidentally infected with mycoplasma (Figure 6). The mycoplasma presence was proved by RT-PCR (Supplementary Figure S1a), by the developed approach (Supplementary Figure S1b), and the MycoAlert Mycoplasma Detection kit (ratio was 39.15218). A very low signal was only observed if less than 5% of the area of the cell nuclei exhibited saturation independently of the fixation protocol (Figure 6, upper images). Although a circa six-fold prolongation of the acquisition time provided a higher signal (Figure 6, lower images), it resulted in saturation of the cell nuclei and made it impossible to resolve the mycoplasmas in this area or in close proximity to it. These results also indicated that there is no or very low impact of the developed procedure on DAPI staining.

We also tested the Hoechst 33,258 instead of DAPI using the slightly adapted protocol described by Young et al. [6] (Supplementary Figure S6). This protocol also provided a very low signal as compared to the developed protocol.

### 3.5. Sensitivity of the Developed Approach

**Figure 7:**
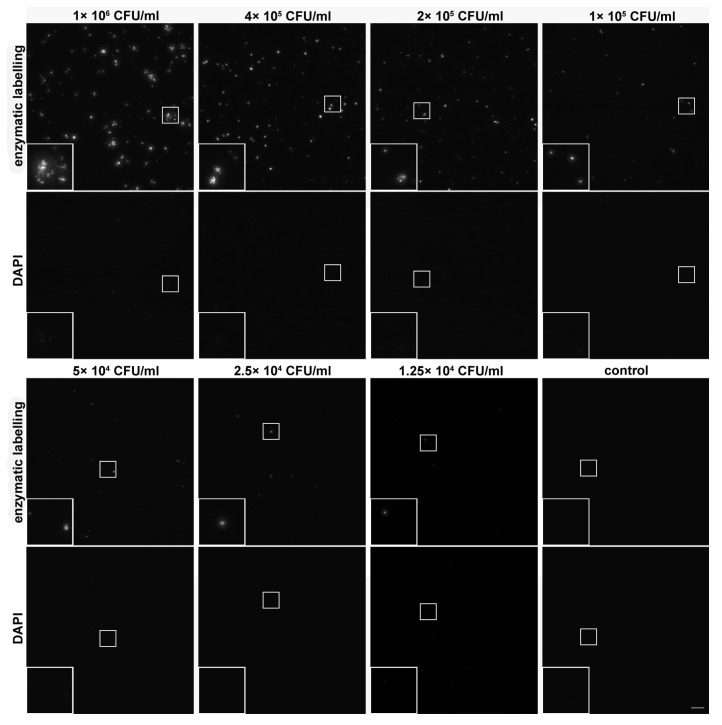
Sensitivity of the optimized assay. The A549 cells were or were not (control) inoculated with the indicated concentrations of *M. hominis*, incubated for 24 h and then processed according to the developed approach using biotin-dUTP for staining of mycoplasmas’ DNA. Mycoplasmas’ DNA was simultaneously stained with DAPI. All images were acquired for 3.404 ms. Scale bar = 10 µm.

To further address the sensitivity of the developed procedure, we analyzed A549 cells inoculated with various concentrations of *M. hominis* (Figure 7). The highest inoculated concentration was 1 × 10^6^ CFU/mL, the lowest concentration was 1.25 × 10^4^ CFU/mL. The A549 cells were then cultivated for 24 h and fixed by formaldehyde. The developed assay, DAPI (Figure 7) and RT-PCR (Supplementary Figure S7) was used for mycoplasma detection.

Although both the area occupied by signal and the signal intensity gradually decreased with the decrease of inoculation concentrations, even the lowest concentration provided discernible areas containing the signal. The higher signal intensity obtained from the samples with the higher inoculated concentrations compared to the lower inoculated concentrations can be probably attributed to the colony formation, resulting in the higher out of focus signal and thus, in the higher signal per camera pixel. All images were acquired for 3.404 ms. The simultaneous detection of DAPI clearly showed that this acquisition time resulted in a much lower signal (Figure 7).

To obtain the significant DAPI signal a more than 10-fold prolongation of the acquisition time was necessary (Supplementary Figure S8). Importantly, if we performed a visual microscopic inspection of the samples, we found that even this high concentration resulted in a very low signal with DAPI. In contrast, the developed method provided a very high and nicely discernible signal that was clearly observable to the naked eye.

The low sensitivity of DAPI is in agreement with the previously published studies showing that the direct DAPI staining is one of the less sensitive approaches. In the case of the low level of infection, it is very difficult to identify correctly mycoplasma positive or negative samples [5,8,25]. Similarly, Jean et al. showed that staining with the other DNA dye, Hoechst, only had a 25% sensitivity compared to the 95.2% sensitivity of qPCR [26]. Our results also clearly showed that even a relatively high concentration of mycoplasmas requires highly sensitive cameras for the valuable signal. No such need was observed in the case of the developed method. The simultaneously performed detection of mycoplasmas by RT-PCR clearly showed that all the tested mycoplasma concentrations are detectable by this method (Supplementary Figure S7).

## 4. Conclusions

The developed technology is based on labeling mycoplasmas’ DNA. Unlike when using DAPI or Hoechst staining, this technology does not intensively stain nuclear DNA, provides the possibility to use the most convenient fluorochrome, and allows more reliable detection of mycoplasma infection in a much lower mycoplasma concentration when compared to DAPI or Hoechst staining. Our results also indicate that the method does not stain bacteria with the cell wall. As the approach can result in the simultaneous staining of mitochondrial DNA, suitable markers such as MTCO2 can be used for improvement of the sensitivity. On the other hand, the staining of mitochondrial DNA can be substantially decreased by the use of freshly fixed cells.

The optimized protocol contains the following steps:

1. Fixation with 2% formaldehyde in 1× PBS (10 min, RT);
2. Washing with 1× PBS (3 times);
3. Permeabilization with 0.2% Triton X-100 in 1× PBS (10 min, RT);
4. Washing with 1× PBS (3 times);
5. Enzymatic labeling of samples (30 min, RT); the solution is composed of 1× buffer for DNA polymerase I, DNA polymerase I (the final concentration is 0.2 U/µL), mixture of dATP, dCTP, dGTP (the final concentration of all nucleotides is 0.05 mM), biotin-dUTP (the final concentration is 12.5 µM);
6. Washing in 25 mM Tris-HCl, pH 7.5 + 150 mM NaCl (3 times);
7. Primary antibody labeling with anti-biotin antibody (dilution - 1:100 in 25 mM Tris-HCl, pH 7.5 + 150 mM NaCl, 30 min, RT). Optionally, an antibody against the mitochondrial marker such as MTCO2 can be added;
8. Washing in 25 mM Tris-HCl, pH 7.5 + 150 mM NaCl (3 times);
9. Secondary antibody labeling with the fluorescently labeled antibody raised against the primary antibody (dilution - 1:100 in 25 mM Tris-HCl, pH 7.5 + 150 mM NaCl, 30 min, RT). If the mitochondrial marker is used, then a secondary antibody against this primary antibody should also be added to this solution. Optionally, DAPI can also be added;
10. Washing in 25 mM Tris-HCl, pH 7.5 + 150 mM NaCl (3 times);
11. Mounting in the mounting medium and observation.

An overview of the advantages and disadvantages of the developed approach and several currently used methods for mycoplasma detection is shown in Table 4.

## 5. Patents

Palacký University Olomouc holds a Czech patent (307415) for the method for determination of mycoplasmas using enzymatic labeling. Names of inventors: A.L., K.K.

## Figures and Tables

**Table 1:** The optimal composition of the enzymatic mixture.

Buffer	Enzyme	Nucleotides	Marker Nucleotide
1× buffer for DNA polymerase I ^1^ (ThermoFisher Scientific)	0.2 U/µL DNA polymerase I (ThermoFisher Scientific)	0.05 mM dATP, dCTP, dGTP (Promega)	12.5 µM biotin-dUTP or digoxigenin-dUTP

^1^ One unit of the used DNA polymerase I catalyzes the incorporation of 10 nmol of deoxyribonucleotide triphosphates (dNTPs) into a polynucleotide fraction (30 min at 37 °C).

**Table 2:** Primers and probe used in a PCR reaction.

Name	Sequence (5′→3′ Direction)	Length
Myco 1 forward primer	GCTGTGTGCCTAATACATGCAT	22 bp
Myco 2 reverse primer	CACCATCTGTCATTCTGTTAACCT	24 bp
Myco P probe	FAM-ATCCGCACAAGCGGTGGAGC-BHQ1	20 bp

**Table 3:** Set-up parameters for the real-time PCR reaction.

Step		Temperature	Time
Initial denaturation		95 °C	15 min
40 Cycles	Denaturation	95 °C	45 s
	Annealing	55 °C	1 min 40 s

**Table 4:** Advantages and disadvantages of several methods used for mycoplasma detection.

Method	Advantages	Disadvantages	References
agar/broth media assay	high sensitivity high specificity	only cultivable mycoplasmas are detected long testing time depends on the quality of the prepared medium and on the handling of samples a microbiology laboratory is necessary	[2,4,6,27,28]
indicator cell culture assay	detects non-cultivable mycoplasma strains high sensitivity	relatively long testing time low specificity mycoplasmas can be hidden by the signal from cell nuclei can produce false positive results due to cell debris	[2,4,6,27,28]
direct DNA staining	fast cheap	low sensitivity low specificity interpretation of results can be difficult for non-trained personnel	[2,4,6,27,28]
PCR	sensitive fast	does not detect all mycoplasma strains can produce false positive or false negative results	[27,28]
biochemical detection using MycoAlert detection kit	fast high specificity simple performance	can produce false positive results caused by bacterial contamination lower sensitivity need of luminometer	[27,28]
the developed assay	fast do not stain bacteria with cell wall various fluorochromes can be used for detection combination with the direct DNA staining and indicator cell lines is possible detection of non-cultivable mycoplasma strains is possible	mitochondria can be stained – mitochondria marker is recommended	

## Supplementary Materials

The following are available online at https://www.mdpi.com/2073-4409/8/12/1510/s1, Figure S1: Results of the verification of mycoplasma infection of Lep cells, Figure S2: Impact of DNase I and Exonuclease III (Exo III) on the signal intensity, Figure S3: Effect of formaldehyde and ethanol fixation on the signal intensity, Figure S4: Detection of mycoplasmas using fluorescein-dUTP, Figure S5: Detection of mycoplasmas in sample containing *E. coli*, Figure S6: Direct Hoechst 33258 staining, Figure S7: Results of RT-PCR of A549 cells inoculated with various concentrations of *M. hominis*, Figure S8: Comparison of the developed approach with the direct DAPI staining.

## References

[1] Thompson C.C., Vieira N.M., Vicente A.C.P., Thompson F.L. (2011). Towards a genome based taxonomy of Mycoplasmas. Infect. Genet. Evol..

[2] Volokhov D.V., Graham L.J., Brorson K.A., Chizhikov V.E. (2011). Mycoplasma testing of cell substrates and biologics: Review of alternative non-microbiological techniques. Mol. Cell Probes.

[3] Halbedel S., Stulke J. (2007). Tools for the genetic analysis of Mycoplasma. Int. J. Med. Microbiol..

[4] Drexler H.G., Uphoff C.C. (2002). Mycoplasma contamination of cell cultures: Incidence, sources, effects, detection, elimination, prevention. Cytotechnology.

[5] Uphoff C.C., Brauer S., Grunicke D., Gignac S.M., MacLeod R.A., Quentmeier H., Steube K., Tummler M., Voges M., Wagner B. (1992). Sensitivity and specificity of five different mycoplasma detection assays. Leukemia.

[6] Young L., Sung J., Stacey G., Masters J.R. (2010). Detection of Mycoplasma in cell cultures. Nat. Protoc..

[7] Baum S.G., Goldman L., Schafer A.I. (2012). Mycoplasma Infections. Goldman’s Cecil Medicine, 24TH EDITION.

[8] Garner C.M., Hubbold L.M., Chakraborti P.R. (2000). Mycoplasma detection in cell cultures: a comparison of four methods. Br. J. Biomed. Sci..

[9] Nikfarjam L., Farzaneh P. (2012). Prevention and detection of Mycoplasma contamination in cell culture. Cell J..

[10] Rottem S., Barile M.F. (1993). Beware of mycoplasmas. Trends Biotechnol..

[11] Uphoff C.C., Drexler H.G. (2014). Eradication of Mycoplasma contaminations from cell cultures. Curr. Protoc. Mol. Biol..

[12] Vankuppeveld F.J.M., Johansson K.E., Galama J.M.D., Kissing J., Bolske G., Vanderlogt J.T.M., Melchers W.J.G. (1994). Detection of Mycoplasma Contamination in Cell-Cultures by a Mycoplasma Group-Specific Pcr. Appl. Environ. Microb..

[13] Benisheva T., Loewer J. (1994). Comparison of Three Methods for the Detection of Mycoplasms in Cell Cultures. Biotechnol. Biotechnol. Equip..

[14] Gigahertz-Optik, Inc. II.6. Spectral Sensitivity of the Human Eye. https://light-measurement.com/spectral-sensitivity-of-eye/.

[15] Spring K.R., Davidson M.W. Concepts in Digital Imaging Technology: Quantum Efficiency. http://hamamatsu.magnet.fsu.edu/articles/quantumefficiency.html.

[16] Andor_Zyla_5.5_and_4.2_PLUS_Specifications. http://www.andor.com/pdfs/specifications/Andor_Zyla_5.5_and_4.2_PLUS_Specifications.pdf.

[17] Carpenter A.E., Jones T.R., Lamprecht M.R., Clarke C., Kang I.H., Friman O., Guertin D.A., Chang J.H., Lindquist R.A., Moffat J. (2006). CellProfiler: image analysis software for identifying and quantifying cell phenotypes. Genome Biol..

[18] Kamentsky L., Jones T.R., Fraser A., Bray M.A., Logan D.J., Madden K.L., Ljosa V., Rueden C., Eliceiri K.W., Carpenter A.E. (2011). Improved structure, function and compatibility for CellProfiler: modular high-throughput image analysis software. Bioinformatics.

[19] Baczynska A., Svenstrup H.F., Fedder J., Birkelund S., Christiansen G. (2004). Development of real-time PCR for detection of Mycoplasma hominis. BMC Microbiol..

[20] Ligasová A., Strunin D., Koberna K. (2013). A New Method of the Visualization of the Double-Stranded Mitochondrial and Nuclear DNA. PLoS ONE.

[21] Ligasová A., Strunin D., Liboska R., Rosenberg I., Koberna K. (2012). Atomic scissors: a new method of tracking the 5-bromo-2’-deoxyuridine-labeled DNA in situ. PLoS ONE.

[22] Minion F.C., Jarvill-Taylor K.J., Billings D.E., Tigges E. (1993). Membrane-associated nuclease activities in mycoplasmas. J. Bacteriol..

[23] Citti C., Blanchard A. (2013). Mycoplasmas and their host: emerging and re-emerging minimal pathogens. Trends Microbiol..

[24] Kapuscinski J., Szer W. (1979). Interactions of 4’, 6-diamidine-2-phenylindole with synthetic polynucleotides. Nucleic Acids Res..

[25] Uphoff C.C., Gignac S.M., Drexler H.G. (1992). Mycoplasma contamination in human leukemia cell lines. I. Comparison of various detection methods. J. Immunol. Methods.

[26] Jean A., Tardy F., Allatif O., Grosjean I., Blanquier B., Gerlier D. (2017). Assessing mycoplasma contamination of cell cultures by qPCR using a set of universal primer pairs targeting a 1.5 kb fragment of 16S rRNA genes. PLoS ONE.

[27] Geraghty R.J., Capes-Davis A., Davis J.M., Downward J., Freshney R.I., Knezevic I., Lovell-Badge R., Masters J.R.W., Meredith J., Stacey G.N. (2014). Guidelines for the use of cell lines in biomedical research. Brit. J. Cancer.

[28] Xu Y., Egan W., Chang A., Webber K. (2005). Mycoplasma In-Process and Lot Release Testing: To PCR or Not to PCR. BioProcess. Int..

