# Supplementary material for: A New Sensitive Method for the Detection of Mycoplasmas Using Fluorescence Microscopy
Source: Cells. 2019 Nov 25;8(12):1510. doi: 10.3390/cells8121510 (PMC6952905; doi:10.3390/cells8121510)
Supplement: Supplementary file 1 [file cells-08-01510-s001.pdf]

Supplementary Figures

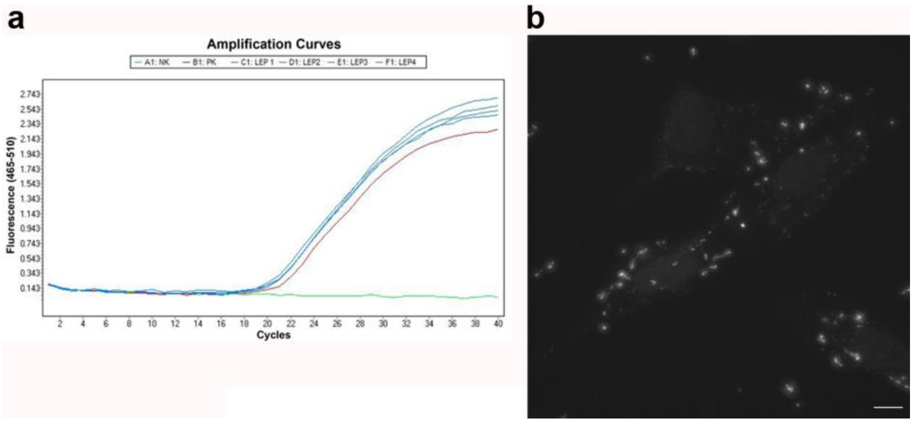

Supplementary Figure S1. Results of the verification of mycoplasma infection of Lep cells

a) Verification of mycoplasma infection in samples with Lep cells using RT-PCR is shown. NK = negative control; PK = positive control, Lep1-Lep4 = samples of lysates from Lep cells.

b) Verification of mycoplasma infection in samples with Lep cells using the developed approach. The exposition time was 13.772 ms. Scale bar = 10 μm.

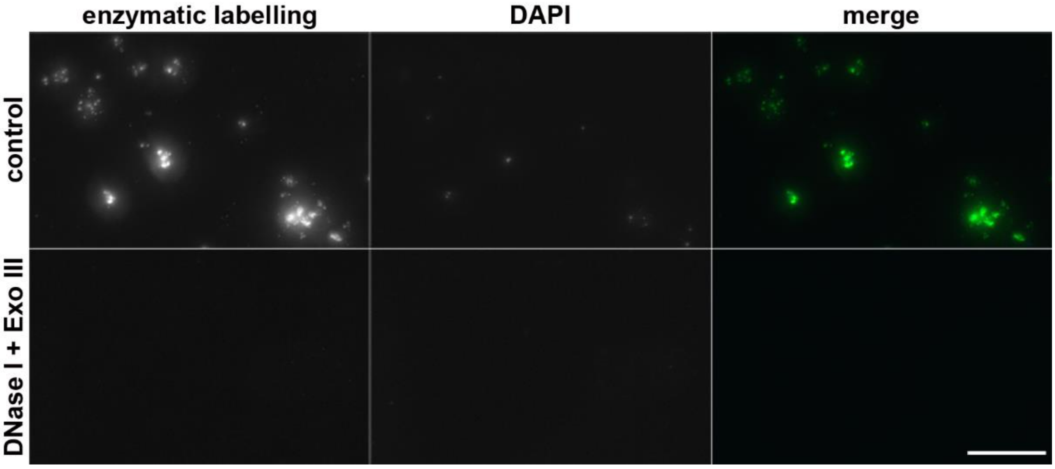

Supplementary Figure S2. Impact of DNase I and Exonuclease III (Exo III) on the signal intensity

The effect of the incubation of the samples in the solution of DNase I (100 U/ml) and Exonuclease III (4 U/μl) on mycoplasmas' DNA is shown. A549 cells inoculated with 1× 10^6 CFU/ml of *M. hominis* were fixed with formaldehyde, permeabilised with Triton X-100 and incubated in the solution of DNA nucleases for 2 hours at 37 °C followed by enzymatic labelling with biotin-dUTP. Biotin-dUTP was visualised using primary and secondary antibodies. Mycoplasmas' DNA was simultaneously stained with DAPI. The control samples were not incubated in the solution of DNA nucleases. The exposition time was 3.404 ms. Scale bar = 10 μm.

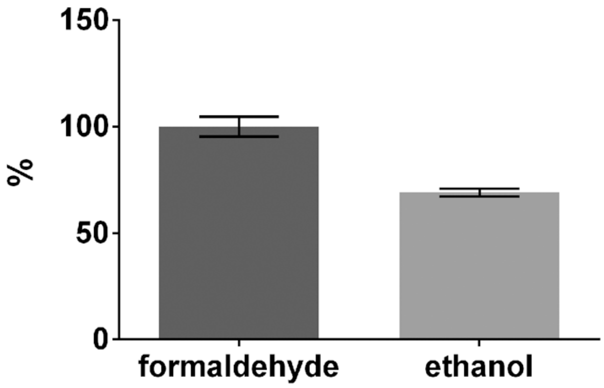

Supplementary Figure S3. Effect of formaldehyde and ethanol fixation on the signal intensity
The graph shows the integrated signal intensity of the labelled mycoplasma in formaldehyde- and ethanol-fixed samples from Figure 2. The signal from ethanol-fixed samples is normalized to the signal measured in formaldehyde-fixed cells which is equal to 100%. The data are shown as the mean ± STD.

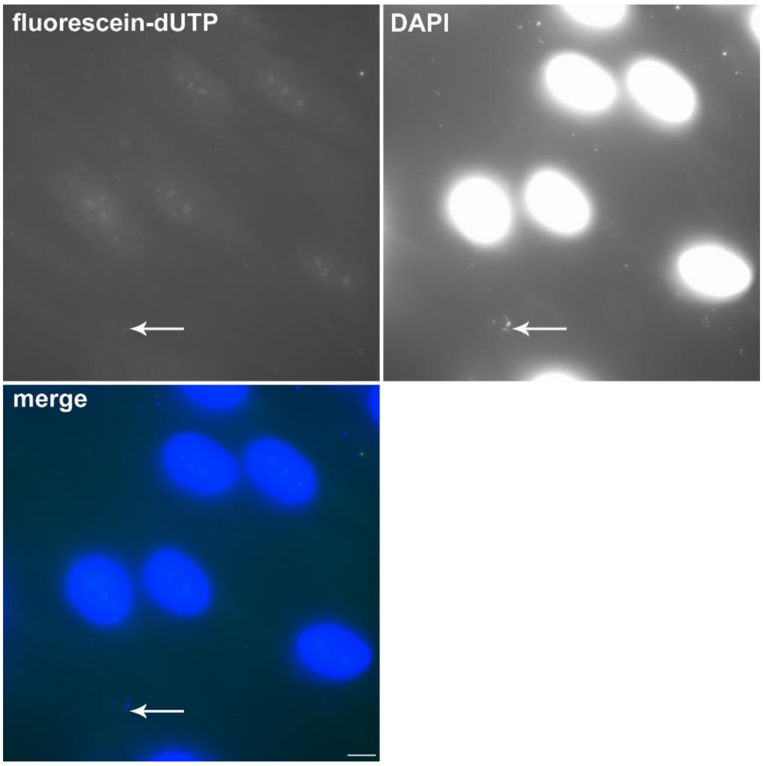

Supplementary Figure S4. Detection of mycoplasmas using fluorescein-dUTP

The samples containing Lep cells accidentally infected with mycoplasma were fixed by formaldehyde, permeabilised by Triton X-100 and mycoplasmas' DNA was detected using enzymatic labelling with Pol I. The enzymatic mixture contained fluorescein-dUTP. The exposition time was 50.012 ms (fluorescein-dUTP) and 80.012 ms (DAPI). The arrows point to the DAPI positive area containing the discernable fluorescein signal. Scale bar = 10 μm.

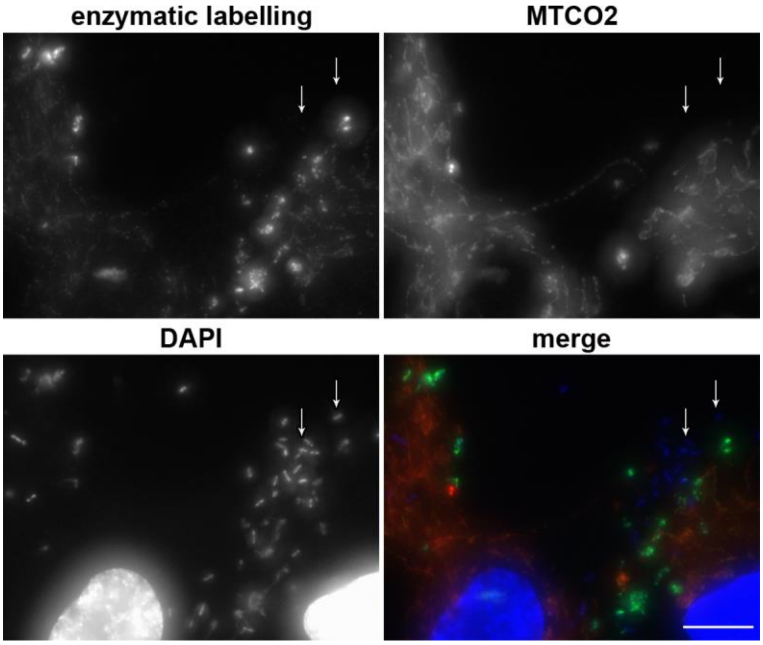

Supplementary Figure S5. Detection of mycoplasmas in sample containing *E. coli*
E. coli deposited on the glass coverslips with Lep cells accidentally infected with mycoplasma were fixed by formaldehyde, permeabilised by Triton X-100 and stained using the developed approach with biotin-dUTP. Mitochondria were stained with anti-MTCO2 primary antibody. Mycoplasmas' DNA was simultaneously stained with DAPI. The images were acquired for 5.012 ms (biotin-dUTP and DAPI) and 4,003 ms (MTCO2). Arrows point to *E. coli* cells. Scale bar = 10 μm.

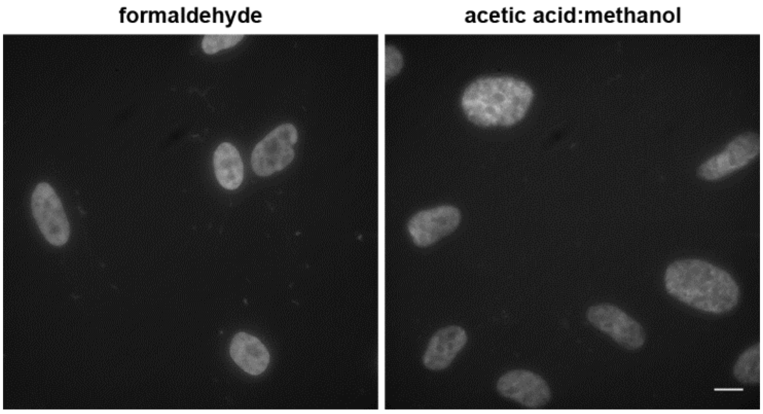

Supplementary Figure S6. Direct Hoechst 33258 staining

The results of the direct Hoechst staining of the Lep cell accidentally infected with mycoplasma. The images were acquired for 181.508 ms (formaldehyde fixation) and 262.412 ms (acetic acid: methanol fixation).Scale bar = 10 μm.

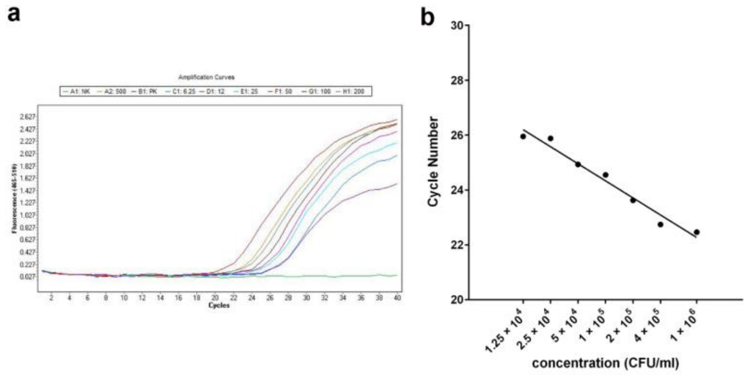

Supplementary Figure S7. Results of RT-PCR of A549 cells inoculated with various concentrations of *M. hominis*.

a) Results of the RT-PCR of A549 cells inoculated with various concentrations of *M. hominis*. 500 = 1× 10^6 CFU/ml; 200 = 1× 10^5 CFU/ml; 100 = 2× 10^5 CFU/ml; 50 = 1× 10^5 CFU/ml; 25 = 5× 10^4 CFU/ml; 12 = 2.5× 10^4 CFU/ml; 6.25 = 1.25× 10^4 CFU/ml; NK = negative control; PK = positive control.

b) The standard curve generated from the crossing points (also known as threshold cycles) of the samples of A549 cells inoculated with the indicated concentrations of *M. hominis* by GraphPad Prism 6 software is shown.

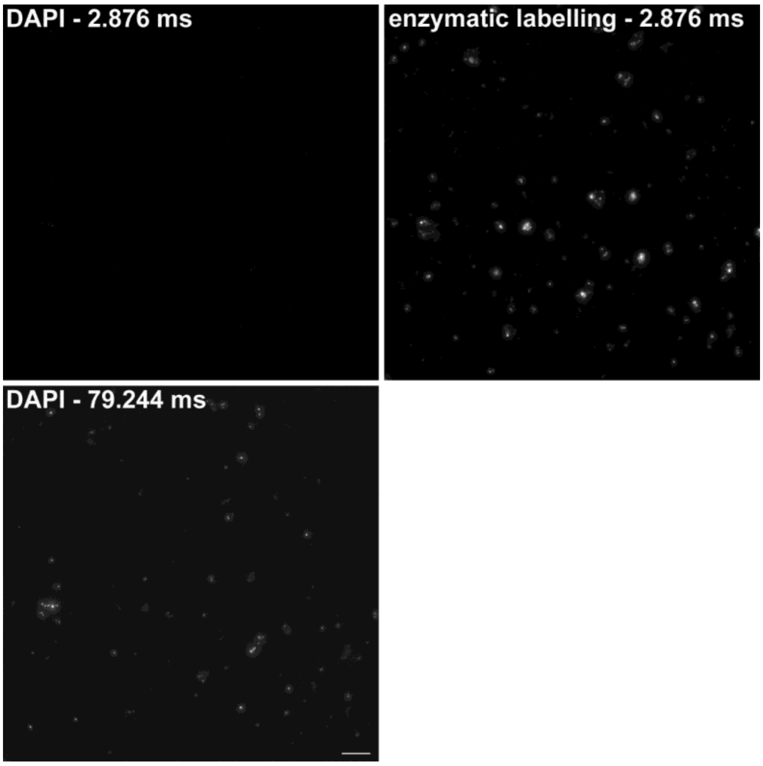

Supplementary Figure S8. Comparison of the developed approach with the direct DAPI staining
Comparison of mycoplasma detection using the direct DAPI staining and the developed approach based on enzymatic labelling (biotin-dUTP). Scale bar = 10 μm.
